# Association of insulin resistance with polycystic ovary syndrome phenotypes and patients’ characteristics: a cross-sectional study in Iran

**DOI:** 10.1186/s12958-023-01160-z

**Published:** 2023-11-25

**Authors:** Leili Rahmatnezhad, Lida Moghaddam-Banaem, Tahereh Behroozi-Lak, Afshin Shiva, Javad Rasouli

**Affiliations:** 1 Department of Reproductive Health and Midwifery, Faculty of Medical Sciences, Tarbiat Modares University, Tehran, Iran; 2 Reproductive Health Research Center, Urmia University of Medical Sciences, Urmia, Iran; 3 Experimental and Applied Pharmaceutical Sciences Research Center, Urmia University of Medical Sciences, Urmia, Iran; 4 Experimental and Applied Pharmaceutical Research Center, Urmia University of Medical Sciences, Urmia, Iran; 5 Department of Epidemiology and Biostatistics, Faculty of Medicine, Urmia University of Medical Sciences, Urmia, Iran; 6 Department of Biostatistics and Epidemiology, School of Medicine, Urmia University of Medical Sciences, Urmia, Iran

**Keywords:** Insulin resistance, Phenotypes, Polycystic ovary syndrome, HOMA-IR

## Abstract

**Background:**

Polycystic ovary syndrome (PCOS) is the most common endocrine disorder in women. This disorder affects 6–15% of women of childbearing age worldwide. It is diagnosed with hyperandrogenism, polycystic ovaries, and chronic anovulation with insulin resistance. This study aimed to assess the prevalence of insulin resistance (IR) in 4 phenotypes of PCOS, and its relationship with demographic, clinical, and paraclinical individual characteristics in a sample of Iranian PCOS patients.

**Methods:**

This particular cross-sectional investigation involved 160 female participants, aged between 18 and 45 years, who were receiving care at gynecology clinics in Urmia, northwestern Iran. All the participants had been diagnosed with PCOS and were categorized into one of four phenotypes. All the participants underwent clinical evaluations, paraclinical assessments, and ultrasound scans. IR was defined as HOMA-IR > 2.5. The statistical significance level was 0.05.

**Results:**

Among the 160 participants, the prevalences of the 4 phenotypes were: A: 83 (51.9%), B: 37 (23.1%), C: 21 (13.1%), and D: 19 (11.9%). IR was detected in 119 participants (74.4%); its rate was significantly different between the 4 phenotypes (*p*-value: 0.008) as A: 62 (74.7%), B: 34 (91.9%), C: 12 (57.1%), D: 11 (57.9%). Linear and logistic regression analyses were performed to control confounding factors. In linear regression, PCOS phenotype, classic phenotype (A&B), economic status, and Hb levels were significantly related to HOMA-IR; in logistic regression Hb levels, exercise, economic status, and PCOS phenotypes were significantly associated with insulin resistance.

**Conclusions:**

The most prevalent PCOS phenotype in this study was A. PCOS phenotypes were significantly related to insulin resistance and HOMA-IR, with the highest levels of insulin resistance and HOMA-IR observed in phenotype B. Determining the phenotype of PCOS may be helpful for better management of PCOS and its associated complications. However, further investigations are recommended in this regard.

## Background

Polycystic ovary syndrome (PCOS) stands as the prevailing endocrine disorder among women of reproductive age. This disorder affects 6–15% of women worldwide [1]. Because the clinical manifestations of PCOS, the frequency of obesity, insulin resistance (IR), and the prevalence of type 2 diabetes vary depending on race and ethnicity, differences in the reported prevalence among the different populations can be justified [2].

In Iran, according to the Rotterdam Standard, this disorder has been reported to have a prevalence of 14.6% [3]. PCOS is usually defined as having: hyperandrogenism, polycystic ovaries, and chronic anovulation; accompanied by insulin resistance, hyperinsulinemia, abdominal obesity, hypertension, and metabolic syndrome [4]. As per the Rotterdam criteria, a diagnosis of PCOS requires the presence of at least two of the following criteria: oligo or anovulation, clinical or biochemical hyperandrogenism, and polycystic ovaries (PCO) [5].

There are four different phenotypes for this syndrome according to the Rotterdam criteria:

(A): Oligomenorrhea + PCO + hyperandrogenism, (B): Oligomenorrhea + hyperandrogenism, (C): Hyperandrogenism + PCO, (D): Oligomenorrhea + PCO. The first two groups (A and B) are called classical phenotypes, and the second two groups (C and D) are called non-classical phenotypes [6].

According to studies, type (A) is the most common form of PCOS phenotype with a prevalence of 60.2%. Elevated testosterone levels, total cholesterol, and LDL cholesterol in phenotype A contribute to an increased susceptibility to cardiovascular diseases, type 2 diabetes, or metabolic syndrome [7]. Comparing anthropometric, hormonal, and metabolic characteristics among four phenotypes of polycystic ovary syndrome based on Rotterdam criteria, showed that phenotype D is closer to ordinary women than other PCOS phenotypes [8].

The pathogenesis of PCOS is a complex combination of genetic and environmental factors. IR and compensatory hyperinsulinemia are seen in 50 to 60% of women with PCOS. In addition, in women with polycystic ovary syndrome, the prevalence of metabolic syndrome is four times higher [9]. Insulin resistance plays a key role in the development of metabolic syndrome, and hyperandrogenism is an important risk factor for metabolic syndrome in PCOS patients [10]. In patients diagnosed with PCOS, insulin resistance is strongly linked to a range of metabolic irregularities, such as heightened aromatase activity and androgen production, alongside impaired progesterone synthesis in granulosa cells [11].

A recent study revealed that insulin resistance in patients with PCOS closely resembled that observed in individuals with pre-diabetes [12]. Women diagnosed with PCOS commonly experience insulin resistance, increased luteinizing follicle-stimulating hormone ratios, abdominal obesity, and infertility [13].

Endocrinologists from the American College of Endocrinology (ACE) and the PCOS association in the congress about assessment and treatment of PCOS and long-term risks in 2015, emphasized the important role of IR in the pathogenesis of PCOS by creating oligomenorrhea and hyperandrogenism with unknown mechanisms. IR is also the cause of many PCOS-related disorders, including obesity, ovulatory failure leading to sub-fertility or infertility, impaired glucose tolerance (IGT), and finally diabetes mellitus (DM). Given the association of IR with numerous disorders, all women with polycystic ovary syndrome should undergo evaluation for IR [14].

In 2011, at the Third Amsterdam Consensus, different phenotypes of PCOS were identified and the classical phenotype (hyperandrogenism and chronic anovulation with or without PCO) was isolated from those with ovulatory disorders and polycystic morphology [15]. Clinical phenotypes show different metabolic risks, and IR is characteristic of the classical phenotype and ovulation [16].

In clinical settings, assessing insulin sensitivity and insulin resistance can be accomplished by performing repeated intravenous glucose tolerance tests to measure sensitivity and by evaluating the homeostatic model for insulin resistance (HOMA-IR) to measure resistance. Healthy and normal weight PCOS women according to National Institute of Health (NIH) criteria have insulin sensitivity and HOMA-IR values in the range of minimum normal and above normal, respectively [17].

Hyperandrogenemia serves as the characteristic biochemical feature of PCOS. Approximately, 80–90% of women experiencing irregular menstrual periods display elevated levels of circulating androgens [18]. PCOS stands as the most prevalent form of androgen excess disorder (AED), and its development is closely linked to insulin resistance. The diagnosis and treatment of insulin resistance in patients with AEDs, including PCOS, spark considerable debate [19]. PCOS manifests in different phenotypes, which not only vary in the range of clinical symptoms but also in the presence or absence and severity of insulin resistance. Studies using euglycemic clamp techniques have revealed that insulin sensitivity is significantly impaired in PCOS patients with the classic or complete phenotype, while it is less severe in those with normoandrogenic or ovulatory phenotypes [20]. Besides insulin-induced androgen secretion, androgens also play a role in causing hyperinsulinemia in women with PCOS [21]. IR contributes to the hyperinsulinemia seen in many women with PCOS, leading to increased androgen production and ovarian dysfunction [22]. Simultaneously, the hormonal imbalances and metabolic disturbances associated with PCOS can further worsen insulin resistance, creating a cycle that perpetuates both conditions [23]. Understanding this relationship is essential for developing effective treatment strategies for individuals with PCOS and IR. Thus, the discovery and understanding of the association between insulin resistance and PCOS may change the management of the condition, cause increased awareness among healthcare providers, and open new avenues for research and targeted therapies. Therefore, this study aims to investigate the prevalence of insulin resistance in different phenotypes of PCOS and its relationship with demographic, clinical, and paraclinical characteristics in a sample of Iranian PCOS patients.

## Methods

Following the STROBE (Strengthening the Reporting of Observational Studies in Epidemiology) guidelines, this study aimed to examine the prevalence and variations of insulin resistance among different PCOS phenotypes.

### Study method and participants

This cross-sectional study was conducted between November 2020 and June 2021, focusing on 160 women aged 18 to 45 years diagnosed with polycystic ovary syndrome (PCOS). The participants were recruited from gynecology clinics in Urmia, located in northwestern Iran. The sampling method used was convenient sampling, enrolling all eligible women until the desired sample size was achieved.

To be included in the study, participants had to meet the following criteria: be between 18 and 45 years of age, have a confirmed diagnosis of PCOS by a gynecologist based on clinical, laboratory, and imaging findings, not be pregnant at the time of enrollment, not be undergoing infertility treatment or taking hormonal medications, and not have taken any medications other than over-the-counter (OTC) painkillers in the last 3 months. Additionally, the interval between the onset of menarche and the study enrollment had to be more than 4 years, and participants should not have had severe underlying diseases like malignancy or thalassemia that could impact menstrual cycles. Furthermore, known endocrinopathies like Cushing’s syndrome, untreated thyroid disorders, and similar conditions were also considered as exclusion criteria.

### Study procedures

All participants underwent an evaluation using a researcher-developed checklist that encompassed demographic, reproductive, and medical characteristics. The checklist covered a range of factors, including age, level of education, occupation, marital status, number of pregnancies, number of abortions, number of children, age of menarche, economic status, physical activity, diet (both general and specific, such as vegetarianism), and medical history. Following enrollment in the study, participants underwent relevant clinical examinations, paraclinical tests, and ultrasounds as part of the evaluation process.

Anthropometric measurements were conducted following a standard protocol and utilizing calibrated tools. Height was measured without shoes against a fixed strip attached to the wall. Weight was measured using a Seka 755 scale with an accuracy of 500 g, and participants wore light clothing and no shoes during the measurement. BMI was calculated as follows: weight (kg) / height^2 (m).

Waist circumference was determined by using a standard measuring tape aligned parallel to the umbilicus, while hip circumference was measured with a meter at the widest part of the hip region. The waist-to-hip ratio (WHR) was then computed by dividing the waist circumference by the hip circumference.

Clinical indications of hyperandrogenism, such as acne, oily skin, and hirsutism, were observed and assessed. A Gynecologist evaluated all clinical findings, including the Ferriman Gallwey score and galactorrhea. For the diagnosis of hirsutism, a thorough evaluation was conducted by taking a medical history and performing a clinical examination, utilizing the Freeman-Galloway rating score to examine nine body areas for the presence of coarse terminal hairs (including the upper lip, chin, chest, upper and lower abdomen, upper arms, and thighs). The severity of hirsutism was rated on a scale of 1 to 4 for each area, and the total scores were summed. Women with a total score equal to or above 8 were considered to have hirsutism [24].

Participants underwent fasting for 10 to 12 hours before venous blood samples were collected to measure the following parameters: Fasting Blood Sugar (FBS), Fasting Insulin Levels (FIL), Total Serum Testosterone, Thyroid Stimulating Hormone (TSH), Thyroxine (T4), Triiodothyronine (T3), Malonaldehyde (MDA), Sex Hormone-Binding Globulin (SHBG), Follicle-Stimulating Hormone (FSH), Luteinizing Hormone (LH), Hemoglobin (Hb), and Platelets (PLT).

FSH, LH, testosterone, insulin and FBS levels were measured by Abnova commercial kit mIU / ml, Cat.N.DE1288 and GmbH, Germany Demeditec Diagnostics, according to the instructions. The levels of MDA in the follicular fluid were assessed using the Thiobarbituric Acid (TBA) method and the TBARS kit (KA1381) manufactured by Abnova, Taiwan.

### Study variables

#### Polycystic ovary syndrome (PCOS)

PCOS was defined according to Rotterdam criteria, by the presence of 2 of these findings: oligo or anovulation, clinical or biochemical hyperandrogenism, and polycystic ovaries (PCO).

##### Insulin resistance (IR)

The assessment was performed using the homeostatic-insulin resistance (HOMA-IR) formula, which is calculated as follows: fasting insulin (mg/dL) × fasting blood glucose / 405 (μU/mL) [25].

HOMA-IR > 2.5 suggested insulin resistance.

##### Body mass index (BMI)

It was determined using the body mass index (BMI) formula, which involves dividing weight by height squared [26].

##### Polycystic ovaries (PCO)

It was characterized as the presence of 10 or more immature follicles in each ovary and/or an ovarian volume exceeding 10 cm^3^ on ultrasound [27].

##### Menstrual disorders/ ovulatory dysfunction (OD)

Included menstrual disorders encompassed amenorrhea, oligomenorrhea, hypomenorrhea, hypermenorrhea, and irregular menstrual intervals, which were determined based on the participants’ medical history. Oligomenorrhea, specifically, was diagnosed when menstrual cycles occurred more than 35 days apart or less than nine times a year [27].

##### Hyperandrogenism

The definition was established by considering the serum levels of male hormones, including Total Serum Testosterone, SHBG, and FAI, along with the presence of clinical signs such as acne, oily skin, hirsutism, and male pattern hair loss. FAI was determined using the formula: [total testosterone] / SHBG × 100 [28].

##### PCOS phenotypes

In the study, PCOS phenotypes were classified among the participants through an assessment of their medical history, clinical examination, and paraclinical tests, utilizing the criteria of Hyperandrogenism (H), Ovulatory Dysfunction (OD), and Polycystic Ovaries (PCO) as follows: Phenotype A: OD + PCO + H; Phenotype B: OD + H; Phenotype C: H + PCO; Phenotype D: OD + PCO [6].

### Data management and analysis

Our model selection strategy aimed to strike a balance between predictive accuracy and model simplicity. We followed a stepwise approach, beginning with a comprehensive model that included all potentially relevant variables. The data were input into the computer and analyzed using IBM® SPSS® software, version 26. A significance level of 0.05 was set for statistical analyses. Categorical variables were compared using the Chi2 test, while quantitative variables in two groups were analyzed using the Student’s t-test or Mann-Whitney U analysis, depending on the normality of the data distribution. For quantitative variables involving more than two groups, the analysis was conducted using the Anova Test or Kruskal-Wallis analysis, depending on the normality of the variables’ distribution. Linear regression analysis was employed to identify factors influencing HOMA-IR.

## Results

Table 1 presents the prevalence of the four phenotypes and the characteristics of the study participants.

**Table 1:** Demographic, clinical and paraclinical characteristics of participants (*n* = 160)

Characteristic (Qualitative)	Grouping	Frequency (Percent)	Characteristic (Quantitative)	Median ± IQR
**Marital status**	Single	91 (56.9%)	Age (years)	24 ± 7
	Married	64 (40%)	Height (cm)	165 ± 6
	Widowed/divorced	5 (3.1%)	Weight (kg)	66.5 ± 14.75
**Job**	Employed	14 (8.8%)	BMI^1^ (kg/m2)	24.61 ± 5.38
	Housewife	57 (35.6%)	age at menarche (years)	12 ± 1
	University student	51 (31.9%)	FSH^2^ (IU/l)	2.73 ± 1.47
	Highschool student	19 (11.9%)	Total testosterone (ng/ml)	0.65 ± 0.58
	self-employment	19 (11.9%)	SHBG^3^ (nmol/l)	31.3 ± 14.58
**Education**	Illiterate	5 (3.1%)	LH^4^ (IU/l)	7.45 ± 5.9
	under diploma	33 (20.6%)	MDA^5^ (μM)	0.64 ± 0.86
	Diploma	29 (18.1%)	FBS^6^ (mg/dl)	80 ± 2
	college education	93 (58.1%)	FI^7^ (μU/dl)	19.65 ± 16.37
			HOMA^8^	3.88 ± 3.26
**Economic status**	poor (expenditure more than income)	44 (27.5%)	Hb^9^ (g/dl)	12.30 ± 0.30
	Good (expenditure less than and equal to income)	116 (72.5%)	TSH (mU/l)	3.20 ± 1.04
**Physical activity**	No (< 90 minutes per week)	66 (41.3%)	T4^11^ (nmol/l)	1.07 ± .24
	Yes (> = 90 minutes per week)	94 (58.8%)	T3^12^ (nmol/l)	1.20 ± 0.85
**PCOS Phenotype**	A	83 (51.9%)	PLT^13^ (N/mm3)	209.50 ± 66.75
	B	37 (23.1%)	FAI^14^	2.04 ± 2.48
	C	21 (13.1%)	WHR^15^	0.81 ± .02
	D	19 (11.9%)		
**Insulin Resistance (IR)**	No (HOMA< 2.5)	41 (25.6%)		
	Yes (HOMA> = 2.5)	119 (74.4%)		
**BMI**	Normal (< 25)	85 (53.1%)		
	Overweight/obese (> = 25)	75 (46.9%)		
**Ovarian cysts**	*N* < 2	7 (4.4%)		
	N > =2	153 (95.6%)		

1. Body mass index, 2. Follicle stimulating hormone, 3. Sex hormone binding globulin, 4. Luteinizing hormone, 5. Malonaldehyde, 6. Fasting blood sugar, 7. Fasting insulin, 8. Homeostasis model assessment of insulin resistance, 9. Hemoglobin, 10. Thyroid stimulating hormone, 11. Thyroxin, 12. Triiodothyronine, 13. Platelets, 14. Free androgen index, 15. Waist-to-hip ratio

Out of the 160 women studied, 83 (51.9%) according to Rotterdam criteria had the highest prevalence with phenotype A. Most women were: single, housewife, university educated. Considering HOMA-IR cut-off point of 2.5, 119 patients (74.4%) had insulin resistance. Eighty-five patients (53.1%) had normal BMI. The median and IQR of the HOMA-IR index in the participants were 3.88 ± 3.26.

The relationship between the HOMA index and demographic, clinical, and paraclinical variables in participants are provided in Table 2.

**Table 2:** Relations between HOMA index with demographic, clinical and paraclinical characteristics of participants (*n* = 160)

**Parameter (quantitative)**	**HOMA**		
	**Correlation coefficient (r)**	***P*** **– value (Spearman’s test)**	
Age (years)	−0.026	0.744	
Height(cm)	−0.007	0.935	
Weight(kg)	− 0.078	0.328	
age at menarche (years)	0.048	0.551	
FSH^1^ (IU/l)	0.028	0.721	
LH^2^ (IU/l)	**−0.174**	**0.027**	
MDA^3^ (μM)	0.119	0.135	
Hb^4^ (g/dl)	**0.244**	**0.002**	
TSH^5^ (mU/l)	--0.052	0.511	
T4^6^ (nmol/l)	−0.041	0.610	
PLT^7^ (N/mm3)	0.100	0.208	
BMI^8^ (kg/m2)	−0.008	0.316	
SHBG^9^ (nmol/l)	−0.151	0.061	
FAI^10^	**0.178**	**0.024**	
WHR^11^	0.053	0.506	
**Parameter (Qualitative)**	**Grouping**	**HOMA-IR** **(Median ± IQR)**	***P*** **– value**
Education	Illiterate (n:5)	6.03 ± 1.98	0.16**
	Under diploma/ Diploma (n:62)	3.61 ± 3.61	
	college education (n:93)	3.85 ± 3.18	
Number of ovarian cysts	< 2 (n:7)	3.65 ± 3.14	0.55*
	> = 2 (n:153)	3.89 ± 3.29	
Job	Housewife (n:57)	6.76 ± 2.88	0.13*
	Employed (n:103)	3.74 ± 3.31	
Phenotype group	Classic (A&B) (n:120)	4.64 ± 3.16	0.001*
	Non classic (C&D) (n:40)	2.58 ± 3.65	
PCOS phenotype^△^	A (n:83)	3.57 ± 3.18	< 0.001**
	B (n:37)	5.73 ± 1.55	
	C (n:21)	2.61 ± 3.80	
	D (n:19)	2.56 ± 3.71	
Marital status	Single (n:96)	3.83 ± 3.40	0.31*
	Married (n:64)	3.90 ± 3.17	
Physical activity	No (< 90 minutes per week) (n:66)	4.96 ± 3.03	0.03*
	Yes (> = 90 minutes per week) (n:94)	3.46 ± 3.37	
Economic group	poor (expenditure more than income) (n:44)	3.92 ± 2.91	0.07*
	Good (expenditure less than income) (n:116)	3.81 ± 2.68	
BMI group (kg/m2)	Normal (< 25) (n:85)	3.94 ± 3.39	0.25*
	Overweight/obese (> = 25) (n:75)	3.63 ± 3.08	

1. Follicle stimulating hormone, 2. Luteinizing hormone, 3. Malonaldehyde, 4. Hemoglobin, 5. Thyroid stimulating hormone, 6. Thyroxin, 7. Platelets, 8. Body mass index, 9. Sex hormone binding globulin, 10. Free androgen index, 11. Waist-to-hip ratio

△ Significance values have been adjusted with pairwise comparisons by the Bonferroni correction for multiple tests. Significant results were: Phenotypes A & b: *P* – value > 0.001, phenotypes C & B: *P* – value > 0.001, phenotypes D & B: *P* – value > 0.001

* Mann-Whitney U Test, ** Kruskal-Wallis

Regarding the relations between HOMA index and demographic, clinical and paraclinical variables in participants, the following parameters were significantly associated with HOMA-IR: LH (*r* = −0.174), Hemoglobin level (*r* = 0.244), FAI (*r* = 0.178), Classic phenotypes (A&B), and phenotype B had the highest levels of HOMA-IR among the PCOS phenotypes with significant *p*-values. The Relations between insulin resistance with demographic, clinical and paraclinical characteristics of participants are shown in Table 3.

**Table 3:** Relations between insulin resistance with demographic, clinical and paraclinical characteristics of participants (*n* = 160)

**Parameter (quantitative)**	**Insulin resistant (*n* = 119)** **Median ± IQR**	**Non- Insulin resistant (*n* = 41)** **Median ± IQR**		***P*** **– value** **(Mann-Whitney Test)**
Age (years)	23 ± 8	24 ± 7		0.44
Height (cm)	164 ± 6.50	165 ± 6		0.92
Weight (kg)	68 ± 14	65 ± 15		0.79
Age at menarche (years)	12 ± 1	12 ± 1		0.73
FSH^1^ (IU/l)	2.73 ± 1.4	2.73 ± 1.45		0.63
LH^2^ (IU/l)	7.9 ± 8	7.2 ± 5.9		0.19
MDA^3^ (μM)	0.5 ± 0.9	0.7 ± 0.8		0.71
Hb^4^ (g/dl)	12.20 ± 0.4	12.40 ± 0.7		**0.04**
TSH^5^ (mU/l)	3.26 ± 0.98	3.10 ± 1.02		0.22
T4^6^ (nmol/l)	1.08 ± 0.28	1.07 ± 0.24		0.38
PLT^7^ (N/mm3)	198 ± 70	217 ± 65		0.40
BMI^8^ (kg/m2)	24.49 ± 5.13	24.60 ± 5.59		0.99
SHBG^9^ (nmol/l)	34 ± 18.55	29.7 ± 15.1		0.32
FAI^10^	1.67 ± 2.23	2.14 ± 2.64		0.08
WHR^11^	0.81 ± 0.01	0.81 ± 0.02		**0.05**
**Parameter (Qualitative)**	**Grouping**	**Insulin resistant (*n* = 119) Frequency (Percent)**	**Non- Insulin resistant (*n* = 41) Frequency (Percent)**	***P*** **– value** **(Chi-Square Tests)**
Education	Illiterate	5 (100%)	0 (0%)	0.25
	under diploma and Diploma	43 (69.4%)	19 (30.6%)	
	college education	71 (76.3%)	22 (23.7%)	
Number of ovarian cysts group	N < 2	7 (100%)	0 (0%)	0.19
	N > =2	112 (73.2%)	41 (26.8%)	
PCOS phenotype	A	62 (74.7%)	21 (25.3%)	**0.008**
	B	34 (91.9%)	3 (8.1%)	
	C	12 (57.1%)	9 (42.09%)	
	D	11 (57.9%)	8 (42.1%)	
Marital status	Single	67 (69.8%)	29 (30.2%)	0.13
	Married	52 (81.3%)	12 (18.8)	
Phenotype group	Classic (A&B)	96 (80%)	24 (20%)	**0.007**
	Non classic (C&D)	23 (57.5%)	17 (42.5%)	
Exercise	No (< 90 minutes per week)	55 (83.3%)	11 (16.7%)	**0.04**
	Yes (> = 90 minutes per week)	64 (68.1%)	30 (31.9%)	
Economic status	Week (expenditure more than income)	41 (93.2%)	3 (6.8%)	**< 0.001**
	Good (expenditure less than income)	78 (67.2%)	38 (32.8%)	
Job	Housewife	47 (82.5)	10 (17.5%)	0.09
	Employed	72 (69.9)	31 (30.1%)	

1. Follicle stimulating hormone, 2. Luteinizing hormone, 3. Malonaldehyde, 4. Hemoglobin, 5. Thyroid stimulating hormone, 6. Thyroxin, 7. Platelets, 8**.** Body mass index, 9. Sex hormone binding globulin, 10. Free androgen index, 11. Waist-to-hip ratio

Hb levels, Exercise, economic status and PCOS phenotypes were significantly associated with IR; phenotype B patients had the highest rate of IR (91.9%). Also, phenotype grouping was significantly related to IR (IR rates in classic and non-classic phenotypes: 80% vs. 57.5%).

Linear and logistic regression analyses were used to control confounding factors as shown in Tables 4 and 5.

**Table 4:** Linear regression analysis for HOMA index in study participants (*n* = 160)

Variable	Beta	*P*-value
Age (years)	0.035f	0.818
number of pregnancies	0.068f	0.053
Economic group	−0.735	**0.018**
Exercise group	−0.594	**0.033**
PCOS phenotype = A	0.090f	0.089
PCOS Phenotype = B	1.986	**< 0.001**
PCOS Phenotype = C	−0.044f	0.185
PCOS Phenotype = D	−0.034f	0.303
FSH^1^(IU/l)	0.028f	0.661
LH^2^(IU/l)	−0.074f	0.209
MDA^3^ (μM)	0.062f	0.174
Hb^4^ (g/dl)	0.366	**< 0.001**
TSH^5^ (mU/l)	−0.029f	0.748
PLT^6^ (N/mm3)	0.158f	0.334
FAI^7^	0.067f	0.112
WHR^8^	−0.564f	0.325
BMI^9^ (kg/m2)	−0.200f	0.299

1. Follicle Stimulating Hormone, 2. Luteinizing Hormone, 3. Malonaldehyde, 4. Hemoglobin, 5. Thyroid Stimulating Hormone,6. Platelets,7. Free Androgen Index, 8. Waist-to-Hip Ratio, 9. Body Mass Index

**Table 5:** Logistic regression analysis for insulin resistance in study participants (*n* = 160)

Variables	*p*-value	Odds Ratio	95% C.I.	
			Lower	Upper
Age (years)	0.141	1.076	0.976	1.186
Number of pregnancies	**0.012**	**2.062**	**1.175**	**3.621**
Economic status (good vs. weak)	**< 0.001**	**0.048**	**0.010**	**0.234**
Exercise (yes vs. no)	0.91	0.440	0.170	1.141
PCOS phenotype	**< 0.001**			
PCOS phenotype (A)	**–**	1	–	–
PCOS phenotype (B)	**0.008**	**6.885**	**1.637**	**28.961**
PCOS phenotype (C)	**0.005**	**0.137**	**0.034**	**0.548**
PCOS phenotype (D)	0.140	0.340	0.081	1.427
FSH^1^(IU/l)	0.691	1.061	0.793	1.418
LH^2^(IU/l)	0.742	1.017	0.919	1.126
MDA^3^ (μM)	0.321	0.690	0.331	1.437
Hb^4^ (g/dl)	0.081	2.665	0.887	8.005
TSH ^5^(mU/l)	**0.038**	**0.652**	**0.435**	**0.977**
PLT^6^ (N/mm3)	0.136	1.009	0.997	1.020
FAI^7^	0.077	1.166	0.983	1.384
BMI^8^ (kg/m2)	0.336	0.938	0.823	1.069

1. Follicle Stimulating Hormone, 2. Luteinizing Hormone, 3. Malonaldehyde, 4. Hemoglobin, 5. Thyroid Stimulating Hormone, 6. Platelets,7. Free Androgen Index, 8. Body Mass Index

A linear regression analysis was performed, using HOMA-IR as the independent variable and potential influential factors as the dependent variables. The results revealed significant associations between HOMA-IR and the PCOS phenotype b, as well as economic status, Exercise group and Hb levels.

In the logistic regression analysis conducted to identify factors influencing insulin resistance (IR), the following factors exhibited statistically significant relationships with IR:

- PCOS phenotype (*p*-value: < 0.001): Patients with phenotype B had over 6.5 times higher risk of IR compared to those with phenotype A (*p*-value: 0.008, OR: 6.885), and in phenotype C, the risk of IR was 0.137 times that of phenotype A.
- Number of pregnancies (*p*-value: 0.012, OR: 2.062): Each pregnancy increased the risk of IR by more than 2-fold.
- TSH (*p*-value: 0.038, OR: 0.652): Each unit increase in Thyroid-Stimulating Hormone reduced IR by 0.652 times.
- Economic status (*p*-value: < 0.001, OR: 0.048): Individuals with good economic status had an IR rate 0.048 times that of those with poor economic status.

## Discussion

This study was conducted on 160 women diagnosed with PCOS in Urmia, a city located in the northwest of Iran. The primary objective was to assess the prevalence of insulin resistance across various phenotypes of polycystic ovary syndrome and investigate its correlation with demographic, clinical, and paraclinical factors among PCOS patients.

In this study, phenotype A emerged as the most prevalent PCOS phenotype, while phenotype D was the least common. The order of prevalence was as follows: A (51.9%) > B (23.1%) > C (13.1%) > D (11.9%).

Vaggopoulos’s study of 266 women in Greece found that the prevalence of phenotype A was higher than that of the other phenotypes [6]. In the study by Sobti et al., it was reported that the incidence of insulin resistance (IR) using the HOMA-IR 2.5 cut-off point was 31%, with the highest prevalence in phenotype A and the lowest in phenotype D [29]. These findings were consistent with our study, where phenotype A was the most common phenotype. In the study by Rashidi et al., the order of phenotypic prevalence was C > B > D > A [30]. Furthermore, in the study conducted by Naderi et al., the order of phenotypic prevalence was C > D > B > A [31]. The observed differences in these findings compared to our study results may be attributed to genetic factors, variations in lifestyle and dietary habits, and discrepancies in the sample size of participants.

Among all PCOS cases in our study, 75% fell within the classical phenotypes (A&B), while 25% were categorized as non-classical (C&D) according to the Rotterdam criteria. In the study by Vaggopoulos et al. [6], the distribution of classical and non-classical phenotypes among PCOS patients was 62.4 and 37.6%, respectively, which closely aligned with our findings.

In the current study, using a HOMA-IR cut-off point of 2.5, insulin resistance was identified in 119 participants (74.4%). This discovery closely paralleled the results of JalaliZand’s investigation, wherein the prevalence of insulin resistance among patients with PCOS was reported as 69.3% [32].

The results of our study revealed varying rates of insulin resistance (IR) among different PCOS phenotypes, with phenotype A showing a rate of 74.7%, phenotype B at 91.9%, phenotype C at 57.1%, and phenotype D at 57.9%. Remarkably, phenotype B exhibited the highest incidence of IR. Accordingly, our study found significant differences in the HOMA index across the four phenotype groups, with the highest HOMA level observed among patients with phenotype B. This finding contrasted with the study conducted by Sobti et al. [29], where the highest IR prevalence was reported in phenotype A and the lowest in phenotype D. These differences may be attributed to genetic factors, lifestyle choices, eating habits, and variations in participant numbers.

Our findings aligned with the results of the study by Pikee et al., which aimed to determine the prevalence of the four PCOS phenotypes and assess their endocrine and metabolic parameters, including IR and metabolic syndrome, in comparison to the control group. In their study, the highest rate of IR was observed in phenotype B [33]. Similarly, in the study conducted by Welt et al., insulin resistance in phenotype B was also found to be elevated, corroborating our findings [34]. Furthermore, our results were in concurrence with the outcomes of the Zamanzadeh et al. study, wherein a notable difference in metabolic syndrome (wherein IR constitutes an important component) was observed among the four PCOS phenotype groups [35].

However, the current study contradicted the findings of Gupta et al., which aimed to explore the correlation between body mass index (BMI), anti-Müllerian hormone (AMH), and IR across different phenotypes of PCOS. Their study reported no significant difference between the various PCOS phenotypes [36].

Based on the findings of this study, the grouping of phenotypes (classical: phenotypes A & B and non-classical: phenotypes C & D) exhibited a statistically significant association with insulin resistance. Additionally, the highest HOMA index values were observed in phenotypes A and B, representative of the classic PCOS phenotype. This observation mirrored the results of the study conducted by Bil et al. [37]. Our results showed that PCOS phenotype had a significant relationship with the HOMA index, which was consistent with the results of the study by Wiweko et al. [38].

Our findings were also in alignment with the study by Vaggopoulos et al., where they concluded that Greek women exhibiting classical phenotypes of PCOS faced a greater risk of metabolic syndrome and impaired glucose homeostasis compared to women with non-classical phenotypes of PCOS [6].

This study also aligns with the research conducted by Diamanti et al., which aimed to analyze the phenotypic spectrum of PCOS and establish the correlation between metabolic, hormonal, and novel ultrasound criteria. The outcomes of their study demonstrated a higher frequency of the classical PCOS phenotype in comparison to the non-classical phenotype [39].

In this study, participants were categorized into two groups: normal (BMI < 25 kg/m^2^) and overweight/obese (≥ 25 kg/m^2^). However, the distribution of insulin resistance in these two groups did not exhibit a significant difference, which contrasts with the findings of Arini et al. [40], who demonstrated a correlation between HOMA-IR and being overweight. This disparity could potentially be attributed to social and cultural factors, dietary habits, and the relatively small sample size within the present study.

Physical activity had a significant reverse relationship with HOMA index in this study. The results are consistent with the findings of Tofighi et al. which showed that serum insulin levels in the experimental group decreased by 44% compared to baseline after 10 weeks of moderate-intensity aerobic exercise [41].

Waist to Hip Ratio (WHR) was significantly associated with insulin resistance. This finding is consistent with the results of Tosi et al. in which there is a relationship between body fat percentage and insulin sensitivity and WHR changes [42]. Since the present study is a cross-sectional study, the observed relationships do not necessarily indicate a causal relationship between the mentioned factors. Another limitation of the study is the convenience sampling method, and the rather small size of the study population, which reduces the generalizability of the findings.

## Conclusions

In our study, PCOS phenotype A exhibited a higher prevalence compared to other phenotypes. Additionally, there were significant variations in insulin resistance among the four PCOS phenotypes, with phenotype B having the highest rate of insulin resistance. Therefore, determining the phenotype of PCOS during its diagnosis will be helpful for better management of PCOS as a rather common gynecologic disorder and its many complications. But surely more thorough investigations are recommended in this regard.

## Data Availability

The dataset used and analyzed during the current study are available from the corresponding author on reasonable request.

## Abbreviations

PCOS: Polycystic ovary syndrome
IR: Insulin resistance
NIH: National Institute of Health
ACE: American College of Endocrinology
IGT: Impaired glucose tolerance
DM: Diabetes mellitus
OTC: Over-the-counter
WHR: Waist-to-hip ratio
FBS: Fasting Blood Sugar
FIL: Fasting insulin levels
TSH: Thyroid stimulating hormone
MDA: Malonaldehyde
TBA: Thiobarbituric acid
HOMA-IR: Homeostatic-insulin resistance
BMI: Body Mass Index
OD: Ovulatory dysfunction
FAL: Free androgen index
FSH: Follicle stimulating hormone
LH: Luteinizing hormone
Hb: Hemoglobin
SHBG: Sex Hormone Binding Globulin
PLT: Platelets
T4: Thyroxine
T3: Triiodothyronine
